# Aerosolizable Marine Phycotoxins and Human Health Effects: In Vitro Support for the Biogenics Hypothesis

**DOI:** 10.3390/md18010046

**Published:** 2020-01-10

**Authors:** Emmanuel Van Acker, Maarten De Rijcke, Jana Asselman, Ilse M. Beck, Steve Huysman, Lynn Vanhaecke, Karel A.C. De Schamphelaere, Colin R. Janssen

**Affiliations:** 1 Laboratory of Environmental Toxicology and Aquatic Ecology, Department of Animal Sciences and Aquatic Ecology, Ghent University, Campus Coupure, Coupure links 653, 9000 Ghent, Belgium; 2 Flanders Marine Institute (VLIZ), InnovOcean site, Wandelaarkaai 7, 8400 Ostend, Belgium; 3 Greenbridge, Ghent University, Wetenschapspark 1, 8400 Ostend, Belgium; 4 Laboratory for experimental cancer research (LECR), Department for Radiation Oncology and Experimental Cancer Research, Ghent University, Campus UZ, De Pintelaan 185, 9000 Ghent, Belgium; 5 Department Health Sciences, Odisee University College, 9000 Ghent, Belgium; 6 Laboratory of Chemical Analysis, Department of Veterinary Public Health and Food Safety, Faculty of Veterinary Medicine, Ghent University, Campus Merelbeke, Salisburylaan 133, 9820 Merelbeke, Belgium

**Keywords:** sea spray aerosols, phycotoxins, oceans and human health, harmful algal blooms, yessotoxins, biogenics hypothesis, mTOR pathway

## Abstract

Respiratory exposure to marine phycotoxins is of increasing concern. Inhalation of sea spray aerosols (SSAs), during harmful *Karenia brevis* and *Ostreopsis ovata* blooms induces respiratory distress among others. The biogenics hypothesis, however, suggests that regular airborne exposure to natural products is health promoting via a downregulation of the mechanistic target of rapamycin (mTOR) pathway. Until now, little scientific evidence supported this hypothesis. The current explorative in vitro study investigated both health-affecting and potential health-promoting mechanisms of airborne phycotoxin exposure, by analyzing cell viability effects via cytotoxicity assays and effects on the mTOR pathway via western blotting. To that end, A549 and BEAS-2B lung cells were exposed to increasing concentrations (ng·L^−1^–mg·L^−1^) of (1) pure phycotoxins and (2) an extract of experimental aerosolized homoyessotoxin (hYTX). The lowest cell viability effect concentrations were found for the examined yessotoxins (YTXs). Contradictory to the other phycotoxins, these YTXs only induced a partial cell viability decrease at the highest test concentrations. Growth inhibition and apoptosis, both linked to mTOR pathway activity, may explain these effects, as both YTXs were shown to downregulate this pathway. This proof-of-principle study supports the biogenics hypothesis, as specific aerosolizable marine products (e.g., YTXs) can downregulate the mTOR pathway.

## 1. Introduction

The (reported) occurrence of harmful algal bloom (HAB) effects to man and environment has increased over the last decades [1,2]. Toxin-producing marine HAB species have received considerable attention since the 1970s. These algae and their toxins, also known as phycotoxins, are best known for the seafood poisoning they may cause. HAB events in the Gulf of Mexico and the Mediterranean have, however, also caused respiratory distress and other health conditions. In these cases, elevated concentrations of brevetoxins [3] and ovatoxins [4] were present in sea spray aerosols (SSAs). Such harmful exposures to aerosolized phycotoxins are rather rare events, requiring a combination of favorable weather conditions for SSA production and a severe toxin-producing HAB. Many toxin-producing algae, however, are non-severe blooming species [5] and most HABs are ephemeral phenomena. The air concentrations of aerosolized phycotoxins (and other marine chemicals) are therefore generally low. It is suggested that these background concentrations could induce positive instead of negative human health effects. Recent research interest into the positive bioactivity of some phycotoxins, like yessotoxin (YTX), also point to their potential therapeutic use [6,7].

Throughout mankind’s history, exposure to seawater and coastal air has been linked to positive health effects. The first records of thalassotherapy date back to the ancient Egyptians, Greeks, and Romans [8,9]. Fairly recently, epidemiological studies have reported a coastal proximity health effect [10,11,12]. Several hypotheses have been raised to explain why coastal residents are—on average—healthier [13]. To date beneficial effects were mainly attributed to psychological mechanisms, based on the premise that humans recognize harmful environments that induce stress [14]. Natural environments would therefore promote wellbeing as they conjure positive feelings, thus reducing stress-related inflammation [15]. This psychological hypothesis is often supplemented with mechanisms related to the coastal environment, such as relaxation, restoration, exposure to sunlight, increased social contact, and additional effects of increased exercise [16]. A second hypothesis attributes these beneficial effects to an improved immunoregulation, which is also referred to as the “old friends” theory. A reduced exposure to microorganisms leads to poorer response to inflammations, and can even lead to psychiatric disorders [17]. The exposure to diverse microbiota present in marine air/aerosols [18] would therefore stimulate the immune system and human health in general. Yet, all of these mechanisms are unlikely to explain long-term physiological benefits according to Rook [19] and Moore [13]. The latter therefore introduced the biogenics hypothesis, which complements the previous hypotheses. He suggested that the coastal health effect is also caused by regular (intermittent) exposure to biogenic compounds and microbiota in sea spray aerosols (SSAs), via an inhibitory activity on the phosphatidylinositol-3 kinase/protein kinase B/mechanistic target of rapamycin (PI3K/Akt/mTOR) cell signaling pathway; hereafter referred to as the mTOR pathway. This is based on the fact that the augmented activity of this kinase pathway is related to multiple pathological conditions (e.g., many cancers, type 2 diabetes, neurodegenerative diseases) [20] while inhibition or downregulation of this pathway has been associated with anti-cancer therapies and positive health effects [20].

In this explorative in vitro study, we aimed to investigate the potential effects of airborne exposure to aerosolizable marine phycotoxins. Adverse effects were evaluated through the assessment of cell viability effects using cytotoxicity assays. The potential downregulation of the mTOR pathway was evaluated via western blotting. Furthermore, given the importance of the mTOR pathway in both normal and cancer cells, we opted to incorporate both a normal (BEAS-2B) and adenocarcinoma (A549) human lung cell line in our study. We first studied the effects of pure phycotoxins. Next, we also examined the effects of homoyessotoxin (hYTX) in a more realistic SSA matrix; this treatment is further referred to as hYTX extract.

## 2. Results

### 2.1. Cell Viability Effects

Two different cell lines were used in this study: Adenocarcinoma alveolar (i.e., A549) and normal bronchial (i.e., BEAS-2B) cells. The dose response curves (DRCs) for both cell lines derived from the different phycotoxins are plotted in Figure S1 (Supplementary Materials). From these results, it is clear that the two cell lines are comparable in sensitivity towards these phycotoxins. Therefore, we focused on the results of the A549 cell line. All data and results for the BEAS-2B cells are available in the Supplementary Materials.

**Figure 1:**
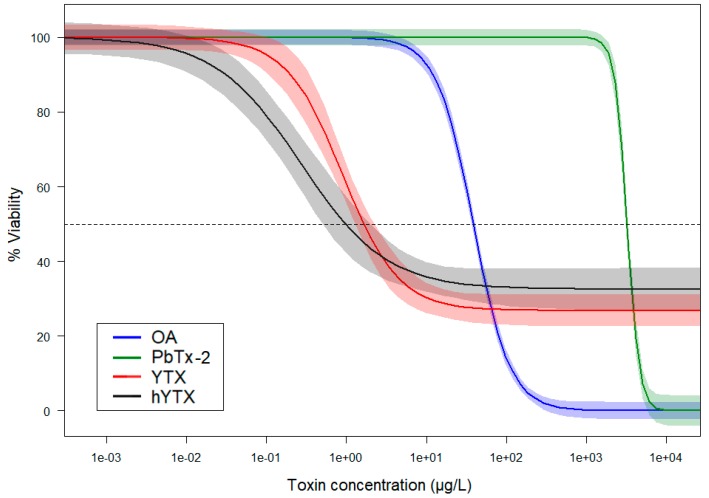
Log-logistic dose response models for four phycotoxins derived from the results of MTT cell viability assays performed on A549 cells over an exposure period of 43 h. The different phycotoxins are okadaic acid (OA), brevetoxin-2 (PbTx-2), yessotoxin (YTX), and homoyessotoxin (hYTX). The light-colored bands are 95% confidence bands. The start cell density was 3000 cells·well^−1^ for all experiments. Parameter estimates (i.e., estimate ± SE) for all dose response models are available in Table S1 under experiment 3-A549 and 9-A549. Note that the DRC models were fitted using all test concentrations, including lower concentrations than the range shown here. To illustrate the goodness of fit of the DRCs, the same graph containing all raw data points is available in the Supplementary Material (Figure S2). The results for domoic acid (DA) are not shown on this graph as there were no effects observed at the highest possible test concentration.

Figure 1 gives a representative overview of the main cell viability results of the 3-(4,5-dimethylthiazol-2-yl)-2,5-diphenyltetrazolium bromide (MTT) assays performed with the A549 cell line and five phycotoxins. To illustrate the goodness of fit of the DRCs, the same graph containing all data points is available in the Supplementary Materials (Figure S2). All toxins, except domoic acid (DA), exerted a negative effect on cell viability. The lowest cell viability effect concentrations were found for YTX and hYTX. Contradictory to the other examined toxins, YTX and hYTX only induced a partial cell viability decrease at the highest test concentrations. Okadaic acid (OA) and brevetoxin-2 (PbTx-2) induced complete mortality, but their effects started at higher test concentrations as compared to YTX and hYTX. A summary of all MTT assays for both cell lines, their derived viability effect concentrations, and the corresponding DRC parameter estimates, is given in the Supplementary Materials (Table S1).

To test the effect of the use of different starting cell densities, part of the MTT assays testing YTX were performed at 8000 cells·well^−1^. This was performed using only the A549 cells, as from previous experimental results, we would not expect a different outcome for the BEAS-2B cells. A comparison between these two starting cell densities (i.e., 3000 vs. 8000 cells·well^−1^) is made in Figure S3 (Supplementary Materials). Again, it is clear that the difference in effects due to a different starting density (3000 vs. 8000 cells·well^−1^) is smaller than the variability between successive experiments.

**Figure 2:**
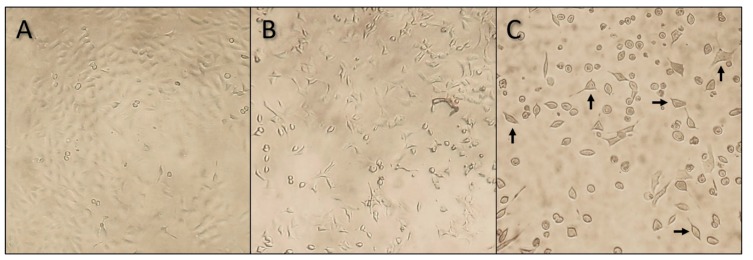
Microscopic images (100× magnification) of A549 cells after a 43-h treatment with pure YTX. The three different images represent three different concentration treatments: (**A**) negative control without YTX, (**B**) 0.5 µg YTX·L^−1^, and (**C**) 400 µg YTX·L^−1^. These visual observations are in line with the cell viability results. In the negative treatment (**A**), a nearly confluent cell culture can be seen. In the mid (**B**) and high (**C**) concentration treatments, cell cultures become less confluent and an increasing number of cells lose their normal cell morphology and deform into round cells. In the high concentration treatment (**C**), a few cells with a more or less normal cell morphology, as indicated with the arrows, are still present.

Following every 43-h exposure period, the cell morphology and density were visually assessed and photographed at 100× magnification. In Figure 2, the effect of YTX on A549 cells is shown at different concentrations. In the negative control, a regular confluent cell culture is observed while in the high concentration treatment (i.e., 400 µg YTX·L^−1^), a much lower cell density and a high frequency of abnormal cell morphology is visible. In the mid-concentration range (i.e., 0.5 µg·L^−1^), somewhat intermediate results are observed, with a slightly lower cell density and a relatively lower amount of normal-shaped cells. These results are complementary with the western blot results (see discussion).

### 2.2. Effects on mTOR Pathway Activity

In exploratory immunoblotting experiments, only YTX exhibited an inhibitory effect on the phosphorylation of S6 ribosomal protein (S6RP), i.e., a downstream phosphorylation target of mTORC1. Therefore, all further experiments were conducted with YTX and analogues of YTX, like hYTX, using wide concentration ranges.

**Figure 3:**
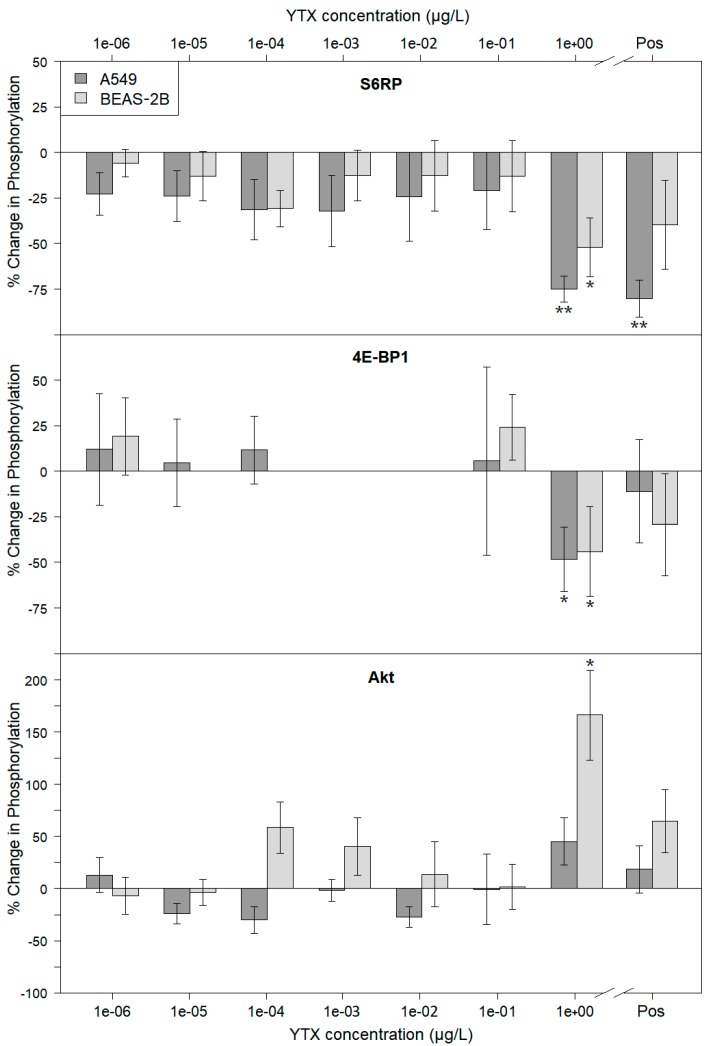
Results of the immunoblotting experiment examining the effects of YTX on mTOR pathway activity. The change (%) in phosphorylation for the three examined phosphorylation markers (i.e., S6RP, 4E-BP1, Akt) was obtained by normalizing the ratio of the phospho-specific and non-phospho-specific responses against the ratio of these measurements of the corresponding negative control treatment. The results are shown for both the A549 and BEAS-2B cell line. Error bars present the standard error (*n* = 3). The positive control treatment, containing 0.3 µM of the known mTOR inhibitor PP242, is indicated as Pos on the *x*-axis. Significant changes in phosphorylation are indicated with asterisk symbols (* *p* < 0.05, ** *p* < 0.01). The results for the 4E-BP1 marker are only shown for the lowest and highest concentration treatments. This is due to distortions on the lower part of the middle lanes (i.e., mid-range concentrations) of the blots (as shown in Figure S4).

In the first main experiment, the effects of YTX on both the A549 and BEAS-2B cell lines were examined. The differential phosphorylation for the examined markers of the mTOR pathway (i.e., protein kinase B (Akt), S6RP, eukaryotic translation initiation factor 4E-binding protein 1 (4E-BP1)) are shown in Figure 3. For each of the two cell lines, a representative example of one of the blots is shown as a cropped non-edited version in the Supplementary Materials (Figure S4). In this experiment (Figure 3), significant effects were observed at the highest concentration (i.e., 1 µg·L^−1^) for all the examined markers. The downstream phosphorylation targets (i.e., S6RP, 4E-BP-1) showed a significant decrease in phosphorylation for both cell lines (i.e., A549 and BEAS-2B) while the phosphorylation of Akt only showed a significant increase for the BEAS-2B cells. An explanation for these variable Akt results is discussed below. Overall, the results are largely comparable for both cell lines and show an inhibitory effect of YTX on the mTOR pathway. This effect (at 1 µg YTX·L^−1^) is even more expressed than the inhibitory effects of the positive control treatment (i.e., known mTOR inhibitor PP242 at 0.3 µM).

**Figure 4:**
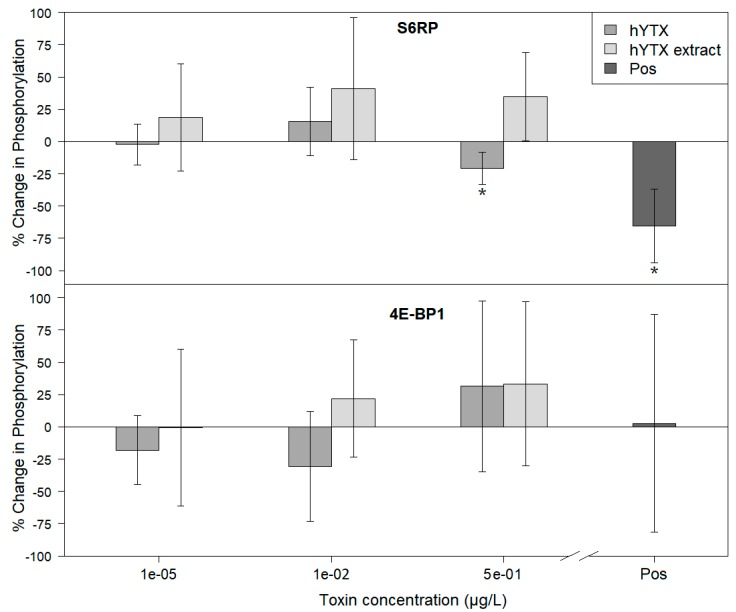
Results of the immunoblotting experiment examining the effects of hYTX and the (experimental SSA) hYTX extract on mTOR pathway activity. Only the A549 cell line was used throughout this experiment. The % change in phosphorylation for the examined phosphorylation markers (i.e., S6RP, 4E-BP1) was obtained by normalizing the ratio of the phospho-specific and non-phospho-specific responses against the ratio of these measurements of the corresponding negative control treatment. Error bars present the standard error (*n* = 3). The positive control treatment, containing 0.3 µM of the known mTOR inhibitor PP242, is indicated as Pos on the *x*-axis. Significant changes in phosphorylation are indicated with asterisk symbols (* *p* < 0.05).

In a subsequent experiment, hYTX and hYTX extract (produced as described below) were dosed to the A549 cells (Figure 4). Due to the limited size of the hYTX extract, the maximum feasible concentration was limited to 0.5 µg·L^−1^ and only A549 cells were incorporated in this experiment. A representative example of one of the blots is shown, as a cropped non-edited version, in the Supplementary Materials (Figure S5). The results demonstrate a significant decrease in phosphorylation for one of the target proteins (i.e., S6RP) for the highest concentration of hYTX. Due to the reduced hYTX concentration (i.e., 0.5 vs. 1 µg·L^−1^) and the complex mixture of the hYTX extract, the results of this experiments are less pronounced. They, however, still support the previous experiments (see discussion).

## 3. Discussion

### 3.1. Cell Viability Effects

Our results (Figure 1 and Table S1) indicate the importance of investigating the effects of aerosolizable marine phycotoxins besides the ones (e.g., brevetoxins, ovatoxins) that are currently known to cause adverse health effects in coastal environments [4,21]. PbTx-2 was the only phycotoxin examined in our study, for which elevated environmental air concentrations and respiratory distress have been reported during toxic HAB (and SSA exposure) [21]. Our cell viability experiments, however, show remarkably lower effect concentrations (i.e., higher toxicity or inhibitory potency) for OA and the two examined YTXs than for PbTx-2. This may indicate a higher pulmonary sensitivity towards these toxins.

Few comparable in vitro experiments have been performed so far. To the best of our knowledge, there are at present no PbTx(-2) effect data for lung cells available in the literature. One of the few records concerning the cell viability effects of PbTx-2 was found for a leukemic T cell line (Jurkat cells). Although Walsh et al. [22] did not report exact effect concentrations, their 48-h EC_50_ value was between 500 and 1000 µg·L^−1^. Wang et al. [23] exposed the A549 cell line (3000 cells·well^−1^) to OA and reported, using MTT cell viability assays, a 48-h EC_50_ value of 34 µg·L^−1^. Based on the cell morphology, they proposed apoptosis as the main cause for the negative cell viability effect. Botana et al. [24] performed sulforhodamine B cell cytotoxicity assays on A549 cells and reported 48-h EC_50_ values for YTX and hYTX of 3.2 and 0.62 µg·L^−1^, respectively. Depending on the exposure period they used in their experiments, the observed effects of YTXs were attributed to apoptosis or autophagy mechanisms [24]. In general, the scarce published data corroborate our experimental results.

YTXs are, in terms of exposure via ingestion (food), considered as the least potent group of phycotoxins. No human intoxications have been reported so far [6]. YTXs are, however, very toxic (LD_50_ of 100–500 μg·kg^−1^) in mice following intraperitoneal injection [25]. The exposure route for these toxins therefore seems of crucial importance in determining toxicity. In our study, YTXs demonstrated very low effect concentrations on lung cells in terms of cell viability. In addition, the shape of their DRCs (Figure 1) differed from the other examined phycotoxins since no complete mortality was obtained at the highest test concentrations. Instead, cell viability (on average) levelled off at around 30%. These findings were also visually confirmed using microscopy (Figure 2), as viable cells were still observed at 400 µg YTX·L^−1^. A decreasing viability trend could, however, still be observed in the high concentration range for YTX and hYTX (Figure S2). If more concentrated YTX standards were available and higher test concentrations were feasible, complete mortality would probably be observed in a higher concentration range. It is, however, clear from the shape of the DRCs that the effect of YTXs we currently encountered in the 0.05 to 100 µg·L^−1^ concentration range cannot be attributed to an adverse outcome pathway leading to direct mortality. Although the mode of action of YTX is not completely understood, YTX has been shown to modify second messenger levels (cAMP), protein levels, and T-lymphocytes, and activate different types of induced cell death [6]. Based on in vitro studies with human lymphocytes, Botana et al. [24] suggested a different mode of action for YTX in tumor cells compared to that in normal cells and therefore highlights its potential as an anticancer drug. They point out that tumor cells undergo apoptosis, paraptosis, and autophagy-induced cell death while in normal cells, only cell proliferation is arrested. Based on this information, one could expect a different cell viability effect in carcinoma A549 cells and normal BEAS-2B cells. This is, however, not confirmed by our cell viability results, which showed little to no difference between the effect on the carcinoma A549 and normal BEAS-2B cell lines. The western blot experiments (discussed below), however, corroborate the findings of Botana et al. [24] as the inhibitory effects on the mTOR pathway were less pronounced for the normal BEAS-2B cells as compared to the carcinoma A549 cells.

### 3.2. Effect on mTOR Pathway Activity

The results from the western blot experiments, summarized in Figure 3 and Figure 4, indicate that both YTXs tested indeed affect mTOR pathway activity. The highest test concentration (i.e., 1 µg·L^−1^) of pure YTX induced a significant downregulation of the phosphorylation of both downstream targets of mTORC1 (i.e., S6RP and 4E-BP1) for both the normal BEAS-2B and carcinoma A549 lung cell lines. Such an inhibitory effect of YTX on mTOR pathway activity was until now only described for human glioma cells exposed to much higher concentrations of 34 and 286 µg·L^−1^ [26]. As discussed in the previous section, such a downregulation of the mTOR pathway leads to autophagy effects, inducing decreased cell proliferation and cell death. The consequent (partial) decrease in cell viability was also observed in the same concentration range (Figure 1), and is therefore expected to be a consequence of this downregulation of the mTOR pathway.

In Figure 3, significantly increased phosphorylation of Akt for the BEAS-2B cells is shown. The feedback loops, activating Akt upon inhibition of mTORC1 phosphorylation, described by O’Reilly et al. [27] and Carracedo et al. [28] can explain these results. The rather smaller inhibitory effects for the downstream phosphorylation targets (i.e., S6RP and 4E-BP1) observed for the BEAS-2B cells, as compared to the A549 cells, can also be linked to these feedback mechanisms. Next, this could also explain the difference in the effect on carcinoma cells (e.g., A549) and normal cells (e.g., BEAS-2B), as suggested by Botana et al. [24] and discussed in the previous section.

The highest test concentration (i.e., 0.5 µg·L^−1^) of pure hYTX induced significant downregulation of S6RP phosphorylation (Figure 4). Although still present, the effects are less strong as compared to the highest test concentration (i.e., 1 µg·L^−1^) of pure YTX in the previous experiment (Figure 3). This can be attributed to the two-fold lower highest test concentration. It is clear that the DRC slope (Figure 1) is sharp in this concentration range and that such a concentration difference may result in a smaller effect. For the hYTX extract treatments, no significant effects were observed. Although the hYTX extract has the same concentration, it may contain (1) other organic molecules or small aerosolizable organic matter that bind or interact with hYTX or (2) other YTX analogues or metabolites with potentially weaker effects that compete with hYTX for molecular binding sites and uptake. Both suggestions imply a decreased bioavailability and lead to weaker effects. This conclusion, concerning the relative weaker effects of this hYTX extract, was also made in our previous study [29]. There, we examined gene expression effects on A549 cells of the hYTX extract, next to pure hYTX and a natural SSA extract. Our previous findings [29], concerning the effects of YTXs on genes related to the mTOR pathway, corroborate our current results and provide additional support for the biogenics hypothesis.

### 3.3. General Discussion and Conclusions

In this study, we aimed to investigate potential human health effects of airborne exposure to phycotoxins that can be present in SSAs. Until now, only a few brevetoxins (PbTxs) and ovatoxins have been detected and quantified in environmental SSAs. Reported PbTx air concentrations during HAB events of *Karenia brevis* ranged from 3 to 180 ng·m^−3^ [3,30]. Although it was not our direct research goal, the production and analysis of the (experimental SSA) hYTX extract proves that other phycotoxins can also aerosolize when present in seawater. Phycotoxin concentrations in SSAs will, among others, be dependent on the density of the toxin-producing algal species in seawater. As YTXs are produced by HAB species that do not bloom to very high densities, their concentrations in the water and consequent SSA phase will never come close to those reported for PbTxs. The lowest reported PbTx concentration in seawater (0.52 µg·L^−1^) [31] that induced negative human health effects is about 57 times higher than the highest potential water concentration for YTX (8.9 ng·L^−1^, calculated in the Supplementary Materials Section S4). Our cell viability experiments, however, suggest that both normal and malignant lung cells are up to 1500 times more sensitive to YTXs as compared to PbTx-2. Furthermore, these cell viability effects of YTXs may be attributed to downregulation of the mTOR pathway. Of course, this does not prove that YTXs (or any other marine chemicals) are the causative agent in SSAs improving the health of coastal populations. To that end, one needs to quantify and assess the ratio of exposure (i.e., air concentrations) and effective in vivo doses of (sea spray) aerosolized chemicals. Our study rather provides proof of principle for the biogenics hypothesis, showing that aerosolizable marine biogenic molecules (e.g., YTXs) can downregulate the mTOR pathway and are potential new therapeutic compounds. Moreover, YTXs can be put forward as interesting target compounds to investigate the marine biogenics hypothesis. Finally, this study also stresses (1) the difference in the sensitivity of different exposure routes, as YTXs are considered to be the least potent phycotoxin group via ingestion exposure and (2) the complexity (e.g., bioavailability) of effects of bioactive substances in realistic environmental (mixture) matrices.

## 4. Materials and Methods

### 4.1. Toxins and Chemicals

Yessotoxin (YTX) and homoyessotoxin (hYTX) were purchased as certified reference material (CRM) from the National Research Council Canada (NRC). All other toxins were analytical-grade products and dissolved, depending on their lipophilic properties, in 50% or 100% methanol or ethanol. Okadaic acid (OA) was purchased from LC Laboratories, domoic acid (DA) at Sigma-Aldrich (Saint Louis, MO, USA), brevetoxin-2 (PbTx-2) from MARBIONC, and Torkinib (PP242)—a known mTOR kinase inhibitor—from MedChem Express (Sollentuna, Sweden).

An experimentally produced SSA extract containing hYTX, in this manuscript referred to as hYTX extract, was generated using a marine aerosol reference tank (MART). This MART was constructed as described by Stokes et al. [32]. The MART tank was filled with artificial seawater and inoculated with *Protoceratium reticulatum* (SCCAP K-1474) at a density of 10^6^ cells·L^−1^. A prior chemical analysis, following the exact procedures described by Orellana et al. [33], showed that this algal strain primarily produced hYTX. As hYTX is structurally similar to YTX, its incorporation and behavior in SSAs are most likely similar. Via the natural bubble bursting process SSAs containing hYTX were generated and sampled on Whatman QM-A quartz microfiber filters at 12 L·min^−1^ during 12 h. The detailed procedure to make extracts of SSA filter samples is described in the Supplementary Materials (Section S1). After extraction, the hYTX concentration in the extract was measured based on the procedures described by Orellana et al. [33]. More details about the filter extraction and chemical analysis are described in the Supplementary Materials (Section S1). As it contains hYTX (with a known concentration) and a mixture of aerosolizable compounds (with unknown concentrations), this hYTX extract represents a more realistic exposure scenario as compared to dosing with pure phycotoxin.

### 4.2. Lung Cell Culturing

To assess the potential effects of airborne exposure to marine phycotoxins, two epithelial lung cell lines were used in a series of in vitro experiments: The adenocarcinoma alveolar basal A549 cell line and the normal bronchial BEAS-2B cell line. As inhibition or downregulation of the mTOR pathway has been associated with anti-cancer therapies and positive health effects, we opted to use both a normal (BEAS-2B) and adenocarcinoma (A549) human lung cell line in our study. As such, these cell lines served as models for (both normal and malignant) exposed lung tissues and, more generally, the lower respiratory tract. The choice to use cell lines originating from the lower respiratory tract is discussed in the Supplementary Materials (Section S5). These historical cell lines were originally set up in collaboration with the LECR at Ghent University. Culture methods and conditions were identical for both cell lines, and only differed in the splitting ratio (see below). The cells were grown in Dulbecco’s modified eagle medium (DMEM), including phenol red, 10% heat-inactivated fetal bovine serum (FBS), 100 U·mL^−1^ penicillin, and 100 µg·mL^−1^ streptomycin, at 5% CO_2_, 37 °C, and a relative humidity of 95% to 100%. Confluent cultures were sub-cultured, using 0.5% Trypsin–EDTA, and split two times per week in a 1/6 (BEAS-2B cells) or 1/8 ratio (A549 cells) or used in the experiments as explained below. Throughout our research, the passage number of all cultured lung cells never exceeded the value of 50.

### 4.3. MTT Cell Viability Assays

To assess the effects of phycotoxins on the viability of cultured lung cells, MTT concentration–response assays were conducted. Upon trypsinization, cells were re-suspended in DMEM (i.e., medium) with the additives as described above, but without phenol red and only 5% of FBS. The cell suspension was diluted and seeded in 96-well plates at 3000 cells per well in 75 µL of medium. Ten hours after incubation, adhered cells were exposed to phycotoxins (i.e., YTX, hYTX, OA, PbTx, DA), added as single substances in 25 µL of medium. Six replicates were used per concentration treatment. Negative, positive, and solvent control treatments were included in each experiment. For the negative control treatments, nothing was added to the medium, and for the positive control treatments, 3% SDS was added. The solvent control contained the same methanol or ethanol concentration as that used in the highest phycotoxin concentration treatments (maximum 2.9% ethanol). After a 43-h exposure period, the cell morphology and confluence were visually assessed and photographed (Figure 2) using an inverted microscope (100× magnification) and 40 µL of 5 mg·mL^−1^ MTT dissolved in Dulbecco’s phosphate-buffered saline (D-PBS; Supplementary Materials Section S2) was added to each well. Next plates were wrapped in aluminum foil and incubated for another 2.5 h. Finally, 100 µL of 10% SDS/0.01 M HCl solution were added and the plates were analyzed, after an overnight incubation at 20 °C, using a spectrophotometer (Multiskan Ascent, Thermo Labsystems, Waltham, MA, USA) at 570 nm and a reference wavelength of 650 nm.

### 4.4. SDS-PAGE and Western Blotting

SDS-PAGE and western blotting were used to assess the mTOR pathway activity of exposed lung cells, allowing us to study the potential positive health effects of phycotoxins as suggested by the biogenics hypothesis. Since the phosphorylation of mTOR has been shown to be a less reliable marker [34,35], we opted to evaluate two downstream phosphorylation targets of mTORC1 (i.e., S6RP, 4E-BP1) instead of the protein itself [34,35]. Next to the two downstream targets of mTORC1, an important upstream kinase in the mTOR pathway (i.e., Akt) was also analyzed via SDS-PAGE and western blotting. The cell exposure setup to analyze the mTOR pathway activity was largely comparable to that used for the MTT viability assays. Cells were seeded in 6-well plates using 3 mL of DMEM (i.e., medium) with the additives as described above, at a final density of 320,000 and 425,000 cells·well^−1^ for the A549 and BEAS-2B cell line, respectively. Ten hours after incubation, phycotoxins (i.e., OA, PbTx, YTX, hYTX, and the hYTX extract) were added as single substances dissolved in 60 µL of methanol. Three replicates were used per phycotoxin treatment. Negative (i.e., 2% methanol) and positive (i.e., 0.3 µM PP242) control treatments were included in every experiment. No solvent control was tested as all treatments, including the negative control, contained the same methanol concentration (i.e., 2%).

After a 43-h exposure, protein cell extracts were prepared and analyzed with SDS-PAGE and western blotting procedures, as discussed in the Supplementary Materials (Section S2). SDS-PAGE was performed with 12% acrylamide precast gels (Bio-Rad, Hercules, CA, USA) and blotting with PVDF membranes (Bio-Rad, Hercules, CA, USA). Immunoblotting was first performed with phospho-specific antibodies directed against phospho-4E-BP1 (Ser65), phospho-S6RP (Ser240/244), and phospho-Akt (Ser473). After analysis with a Chemidoc Imaging CCD-camera (Bio-Rad), antibodies were stripped from the membranes and the procedure was repeated with non-phospho-specific antibodies directed against 4E-BP1, S6RP, and Akt. All primary antibodies were rabbit antibodies used at a 1/1000 dilution. The secondary antirabbit immunoglobuline G (IgG), HRP-linked antibody was used at a 1/3000 dilution. All antibodies were purchased from Cell Signaling Technologies.

### 4.5. Data Processing, Regression, Statistics, and Data Archiving

The raw data of the MTT assays consisted of absorbance measurements. Using the RStudio software, cell viability dose response curve (DRC) models were fitted to these data. The exact procedure of how the raw absorbance data were transformed to cell viability values and how the final DRC models were made is explained in the Supplementary Materials (Section S3). Consequently, the effect concentrations for a 10% (EC_10_) and 50% (EC_50_) decrease in cell viability were derived from the DRC models.

The raw data of the western blot analyses were image-derived chemiluminescent response values. We were specifically interested in the down or upregulation in the phosphorylation of the three target proteins. To that end, the ratio of the phospho-specific and respective non-phospho-specific signals was divided by the ratio of these measurements of the corresponding negative control (on the same blot) and subtracted with 1. In this way, for all treatments and examined proteins, a down (<0%) or upregulation (>0%) in phosphorylation, as compared to the negative control (=0%), was determined. Data within each treatment were checked for normality with a Shapiro–Wilk test (*p* > 0.05). Subsequently, one-sample t-tests were performed to check for significant differences (*p* < 0.05) with the negative control (=0%).

The raw data of the performed MTT and western blot experiments (Raw data—Van Acker (2020) Aerosolizable Marine Phycotoxins and Human Health Effects—In Vitro Support for the Biogenics Hypothesis.zip) are available on the marine data archive (MDA) of the Flanders Marine institute under “VLIZ External datasets— Public/UG/”.

## Supplementary Materials

The following are available online at https://www.mdpi.com/1660-3397/18/1/46/s1, Figure S1: Comparison of the cell viability effects on two different types of epithelial lung cells (i.e., A549 and BEAS-2B) using the dose response model fits of multiple experiments. The 3 different phycotoxins tested, shown from left to right, are brevetoxin-2 (PbTx-2), okadaic acid (OA) and yessotoxin (YTX). These MTT assays were performed over an exposure period of 43 h with a start cell density of 3000 cells·well^−1^ for all experiments shown here, except for the experiments on YTX for which 8000 cells·well^−1^ were used. Note that the DRC models were fitted using all test concentrations, including lower concentrations than the range shown here, Figure S2: Log-logistic dose response curve (DRC) models fitted to the experimental data of MTT cell viability assays performed on A549 cells over a exposure period of 43 h. The five different phycotoxins tested are okadaic acid (OA), brevetoxin-2 (PbTx-2), yessotoxin (YTX), homoyessotoxin (hYTX) and domoic acid (DA). The start cell density was 3000 cells well^−1^ for all experiments. The parameter estimates (i.e., estimate ± SE) for all dose response model fits shown here are available in Table S1 under experiment 3-A549 and 9-A549. For DA the data points are shown without a DRC as there were no effects observed at the highest possible test concentrations, Figure S3: Comparison of the cell viability effects of yessotoxin (YTX) at different start cell densities of 3000 and 8000 cells·well^−1^, using the log-logistic dose response model fits of multiple experiments. These MTT assays were performed on A549 cells over a exposure period of 43 h. The parameter estimates (i.e., estimate ± SE) for all model fits shown here are available in Table S1 under experiment 6-A549, 7-A549, 8-A549 and 9-A549, Table S1: Summary of the effect concentrations and log-logistic dose response models parameter estimates for all performed MTT cell viability experiments. 10% and 50% effect concentrations are reported as 43 h-EC_10_ and 43 h-EC_50_ values. The results shown here are for two different types of epithelial lung cell lines (i.e., A549 and BEAS-2B cells) and for four different phycotoxins. The four different phycotoxins are okadaic acid (OA), brevetoxin-2 (PbTx-2), yessotoxin (YTX), and homoyessotoxin (hYTX). Since the exposure to domoic acid (DA) induced no viability effects, no data of these experiments are shown. The minimum estimate values for OA and PbTx-2 are not relevant (NR) as the these are all 0%. Only in experiment 9, the effect of hYTX was tested. For other experiments no data is available (NA) for hYTX, Figure S4: Cropped, non-edited versions of blots related to the experiment shown in Figure 3. For both (A) the A549 and (B) BEAS-2B cell line, a representative example of one of the blots is shown. A darker band indicates a stronger chemiluminescent signal, Figure S5: Cropped, non-edited versions of a representative blot for the experiment shown in Figure 4. A darker band indicates a stronger chemiluminescent signal.
