# Supplementary material for: Aerosolizable Marine Phycotoxins and Human Health Effects: In Vitro Support for the Biogenics Hypothesis
Source: Mar Drugs. 2020 Jan 10;18(1):46. doi: 10.3390/md18010046 (PMC7024199; doi:10.3390/md18010046)
Supplement: Supplementary file 1 [file marinedrugs-18-00046-s001.pdf]

Supplementary

Aerosolizable Marine Phycotoxins and Human Health Effects: In Vitro Support for the Biogenics Hypothesis

Emmanuel Van Acker [1,*], Maarten De Rijcke [2], Jana Asselman [1,3], Ilse M. Beck [4,5], Steve Huysman [6], Lynn Vanhaecke [6], Karel A.C. De Schamphelaere [1] and Colin R. Janssen [1]

1. Membrane Filter Extraction and Chemical Analysis of the hYTX Extract

Sea spray aerosols (SSAs) were extracted from membrane filters following the protocol described in Baelus [36]. In brief, filters were cut in two equal pieces. One of these halves was eluted with 10 mL methanol followed by sonication. These elution steps were repeated a second time with 3 mL methanol. Then, the combined eluent was filtered over a 0.2 µm PTFE filter. This was followed by evaporation of the sample under a gentle nitrogen gas stream at 40 °C until ±250 µL remained. Half of this extract (mass based) was stored at −20°C for further experimental use, with the other half the hYTX concentration was determined using the analytical techniques described in Orellana *et al.* [33]. This method is based on ultra-high-performance liquid chromatography coupled to high-resolution Orbitrap mass spectrometry (UHPLC-HR-Orbitrap MS). The hYTX concentration (30.6 ng·mL^{-1}) in the final SSA extract was quantified using a calibration curve (0; 10; 20; 30; 40 ng·mL^{-1}; R^2 = 0.998), which was made by spiking hYTX standard (certified reference material purchased at National Research Council Canada) in blanc filter extracts. At the same time, the limit of detection (LOD; 0.28 ng·mL^{-1}) and limit of quantification (LOQ; 0.92 ng·mL^{-1}) of the method used were determined from a lower range calibration curve (0; 0.25; 0.5; 0.75; 1.00 ng·mL^{-1}; R^2 = 0.961).

The other corresponding half of the filter was eluted with 5mL of 0.14 M HNO_3 followed by a vortex, sonication and centrifugation step. These elution steps were repeated a second and third time. Then, the joined eluent was filtered over a 0.45 µm Supor filter and analysed for the Na^+ content using inductively coupled plasma optical emission spectrometry (ICP-OES). From the ICP-OES results the "size" or total amount of collected aerosol could be determined. In this way the Na^+ concentrations in the different hYTX extract treatments can be calculated. The highest concentration treatment of the hYTX extract (0.5 µg hYTX·L^{-1}) contains 2.8 µg Na^+·well^{-1}.

2. SDS-PAGE and Western Blotting Procedures

Following the 43-h exposure period, the medium was removed and the cell monolayer was washed with ice cold D-PBS (137 mM NaCl; 8.1 mM Na_2HPO_4; 2.6 mM KCl; 1.5 mM KH_2PO_4). Cells were scraped from the surface, resuspended in D-PBS and transferred to a sterile Eppendorf tube. A second and third washing step was performed using 1 mL ice cold D-PBS and centrifugation (4 °C at 300× g), at every turn. The cell pellet was subsequently resuspended in 100 µL lysis buffer and put on ice for 15 min. The lysis buffer consisted out of 99% Totex buffer (20 mM HEPES/KOH pH 7.9; 0.35 M NaCl; 1 mM $MgCl_2$; 20% glycerol; 1% Triton X-100; 0.5 mM EDTA; 0.1 mM EGTA) and 1% protease & phosphatase inhibitor cocktail (Halt, Thermo Fisher Scientific). Cell lysates were clarified by centrifugation for 15 min at 20,000× g and 4 °C. Protein concentrations were subsequently determined using Bradford analysis. Related samples were diluted to the same protein concentration, using additional 1× Laemmli on top of the 4× Laemmli loading buffer (250 mM Tris/HCl pH 6.8; 8% SDS; 40% Glycerol; 0.008% Bromophenol blue; 20% mercaptoethanol). Samples with a considerable lower protein concentration as compared to the average, were taken out of consideration for the normalisation of the protein concentrations.

3. Data Transformation to Finally Produce Cell Viability Dose Response Curve Models

Using the DRC package 3.0-1 [37] in RStudio software, a 4-parametric log logistic dose response curve (DRC) model was first fitted to the absorbance data. If the highest concentration treatments

induced complete cell mortality, based on microscopic observations and the absorbance data, the upper (i.e., 100% viability) and lower limits (i.e., 0% viability) of these first DRC model fits were used to transform the absorbance data to relative (%) cell viability values. In the cases where the highest concentration treatments only showed partial mortality effects, the positive control treatment was used instead as a lower normalisation limit (i.e., 0% viability). With this transformed data the final cell viability DRC models were made using 2- or 3-parametric log logistic models. Consequently, effect concentrations for 10% (EC_{10}) and 50% (EC_{50}) decrease in cell viability were derived from the final DRC models.

4. Calculating the Highest Potential YTX Water Concentration

Currently, literature reports yessotoxin concentrations in shellfish and other marine organisms but not in water nor in SSAs. Therefore, we calculated the highest potential YTX water concentration. First, reported cell densities of *Protoceratium reticulatum* during a bloom in the marine environment can rise up to 400–700 cells·L^{-1} [1,2]. Second reported yessotoxin production for *P. reticulatum* strains can go up to 28.6 to 33.6 pg[EVA1]·$cell^{-1}$ [3,4]. Third, up to 38% of the produced YTXs are found extracellular and dissolved in the surrounding water [3]. By using these data we calculated a maximum potential water concentration for YTXs of 8.9 ng·L^{-1} (= 700 cell·[EVA2]L^{-1}, 33.6 pg·[EVA3]$cell^{-1}$, 38%).

5. Importance of Lower Respiratory System for SSA Exposure

Cheng et al. [3] determined the SSA deposition in the upper (75–84%) and lower respiratory tract (2–6%) based on the size distribution of SSAs and the ICRP 66 lung model. Based on these deposition rates, one could ask the question why not to examine effects on cell lines from the upper airways (i.e., extrathoracic region) instead of the lower airways (i.e., tracheobronchial and pulmonary region). However, as described in reference [13], a large fraction of the (solid) particles that are deposited in the upper airways associate with the mucus membranes, are transported to the laryngopharynx and become ingested. The human health effects of SSA exposure by gastrointestinal uptake would make an interesting topic in itself, but is outside the scope of this study. A small SSA fraction (2–6%) is deposited in the lower airways. According to (new) reference [42], the clearance for the small particles penetrating in the lower airways is much slower and soluble chemicals can dissolve in the epithelial lining fluid. Despite low deposition rates of SSAs in lower airways, harmful biogenic chemicals are able to induce lower airway symptoms (e.g. cough, wheezing, chest tightness in the case of PbTx exposure), illustrating their potential for significant human health effects. Further evidence for the penetration of small sized aerosols into the lower airways can be found in the medical world. There are numerous medical therapies using aerosol devices to deliver miscellaneous drugs to treat both pulmonary and non-pulmonary diseases [43]. This drug delivery is also an attractive route to treat non-pulmonary diseases due to fast absorption by the massive alveolar surface area, the abundant vasculature and thin air–blood barrier [43]. Regarding all the above, the relative importance of SSA exposure in the lower airways is much more important than one would expect from the deposited SSA fraction (2–6%) [3].

Additional supportive Figures and Tables

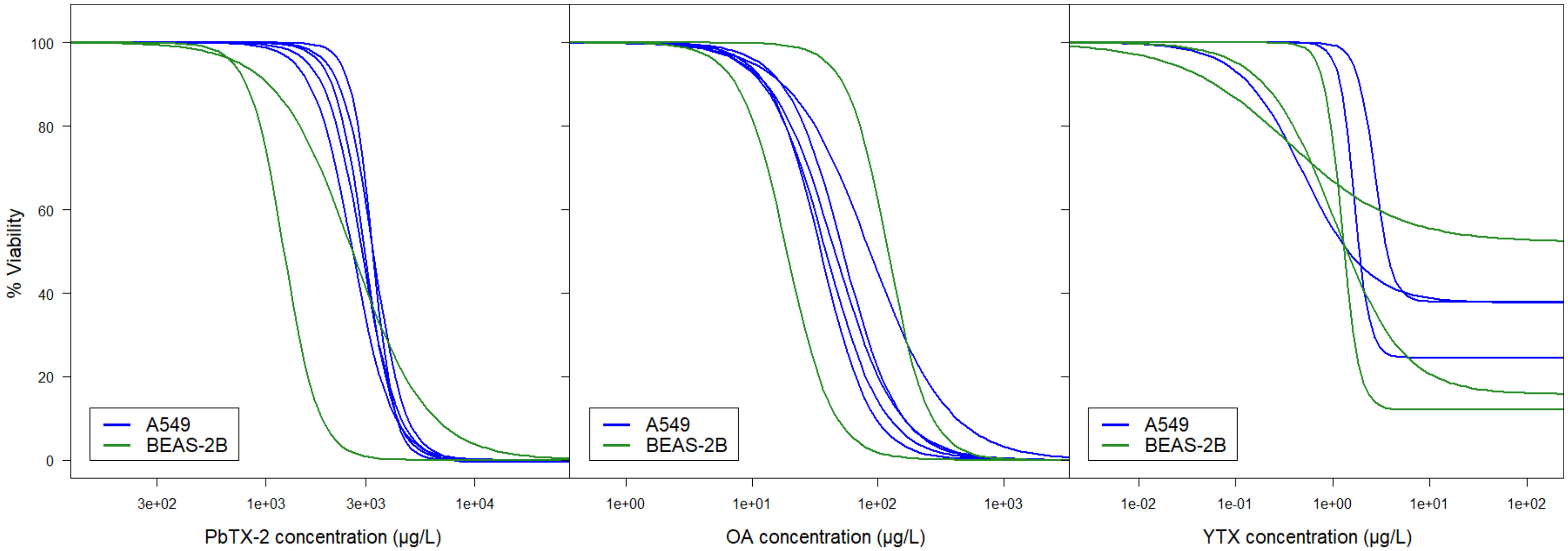

Figure S1: Comparison of the cell viability effects on two different types of epithelial lung cells (i.e., A549 and BEAS-2B) using the dose response model fits of multiple experiments. The 3 different phycotoxins tested, shown from left to right, are brevetoxin-2 (PbTx-2), okadaic acid (OA) and yessotoxin (YTX). These MTT assays were performed over an exposure period of 43h with a start cell density of 3000 cells·well^{-1} for all experiments shown here, except for the experiments on YTX for which 8000 cells·well^{-1} were used. Note that the DRC models were fitted using all test concentrations, including lower concentrations than the range shown here.

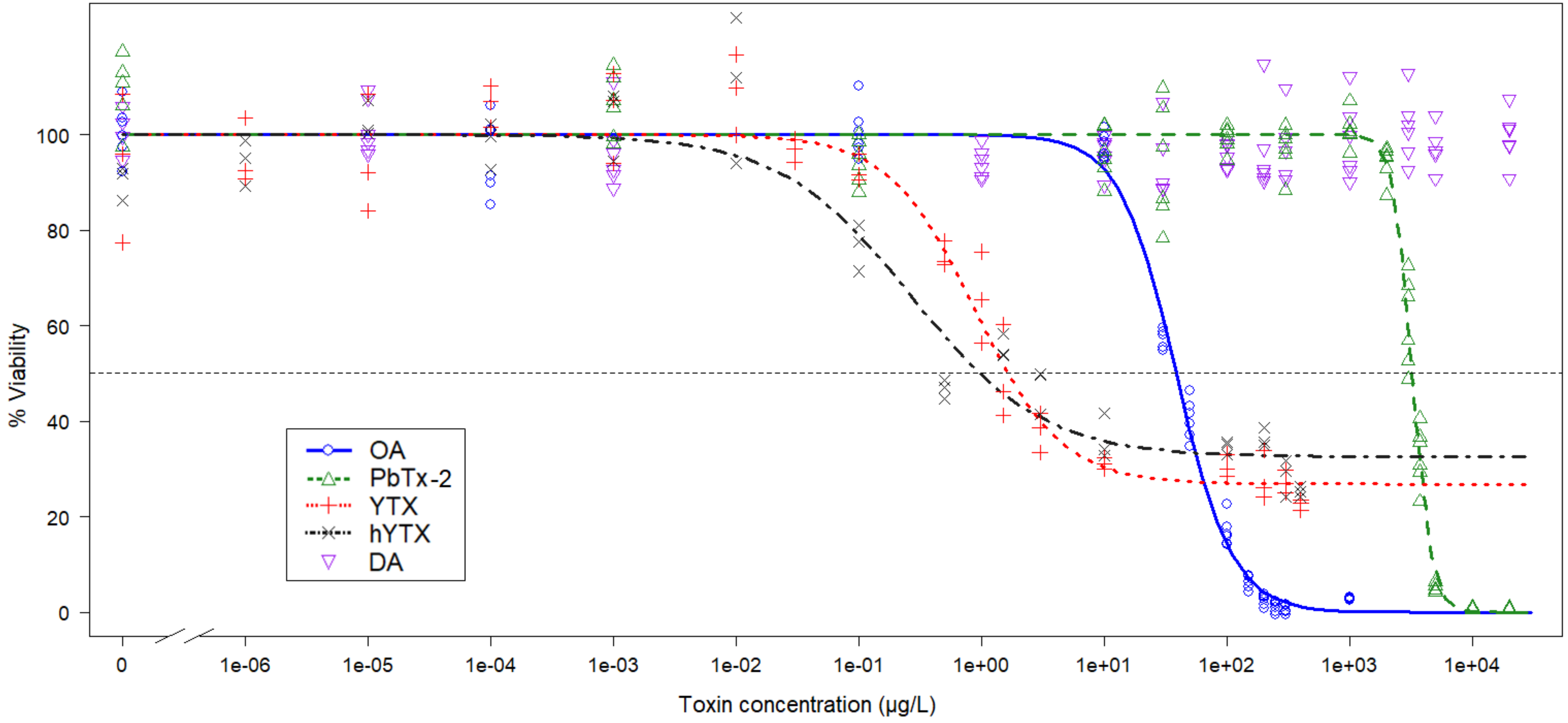

Figure S2: Log-logistic dose response curve (DRC) models fitted to the experimental data of MTT cell viability assays performed on A549 cells over a exposure period of 43h. The five different phycotoxins tested are okadaic acid (OA), brevetoxin-2 (PbTx-2), yessotoxin (YTX), homoyessotoxin (hYTX) and domoic acid (DA). The start cell density was 3000 cells·well^{-1} for all experiments. The parameter estimates (i.e., estimate ± SE) for all dose response model fits shown here are available in Table S1 under experiment 3-A549 and 9-A549. For DA the data points are shown without a DRC as there were no effects observed at the highest possible test concentrations.

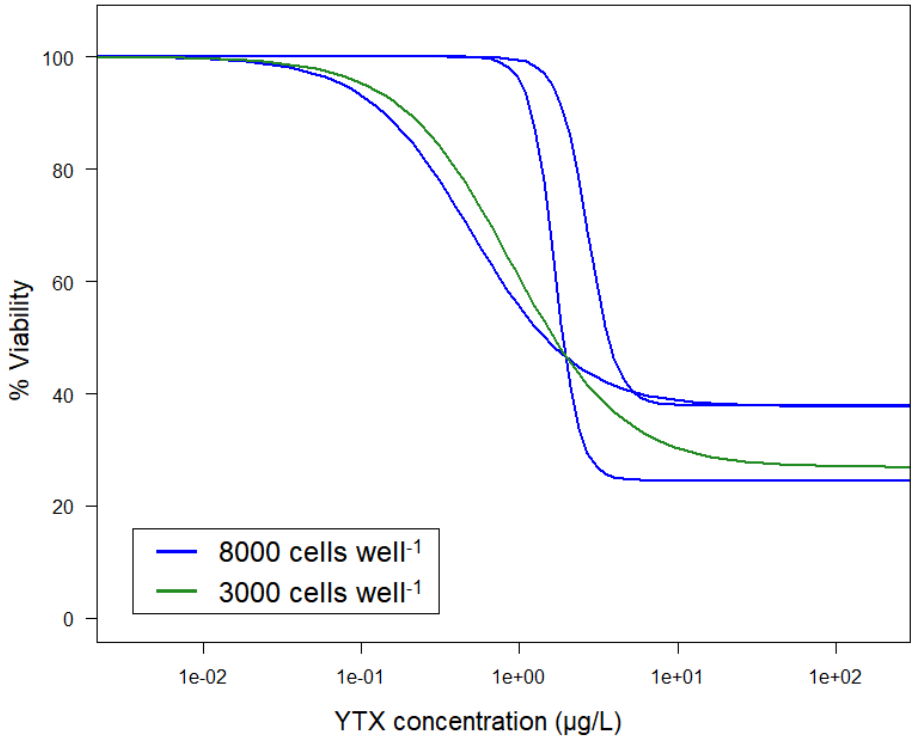

Figure S3: Comparison of the cell viability effects of yessotoxin (YTX) at different start cell densities of 3000 and 8000 cells·well^{-1}, using the log-logistic dose response model fits of multiple experiments. These MTT assays were performed on A549 cells over a exposure period of 43h. The parameter estimates (i.e., estimate ± SE) for all model fits shown here are available in Table S1 under experiment 6-A549, 7-A549, 8-A549 and 9-A549.

Table S1: Summary of the effect concentrations and log-logistic dose response models parameter estimates for all performed MTT cell viability experiments. 10% and 50% effect concentrations are reported as 43-h-EC_{10} and 43-h-EC_{50} values. The results shown here are for two different types of epithelial lung cell lines (i.e., A549 and BEAS-2B cells) and for four different phycotoxins. The four different phycotoxins are okadaic acid (OA), brevetoxin-2 (PbTx-2), yessotoxin (YTX), and homoyessotoxin (hYTX). Since the exposure to domoic acid (DA) induced no viability effects, no data of these experiments are shown. The minimum estimate values for OA and PbTx-2 are not relevant (NR) as the these are all 0%. Only in experiment 9, the effect of hYTX was tested. For other experiments no data is available (NA) for hYTX.

**Experiment & cell type**	**Start cell density (cells·well^{-1})**	**43-h-EC_{10} (μg L^{-1})**		**43-h-EC_{50} (μg L^{-1})**		**Inflection point (μg L^{-1})**		**Slope**		**Minimum (%)**	
		**OA**	**PbTx-2**	**OA**	**PbTx-2**	**OA**	**PbTx-2**	**OA**	**PbTx-2**	**OA**	**PbTx-2**
1 - A549	3000	17.2 ± 1.1	1889 ± 167	52.8 ± 1.6	2884 ± 64			1.96 ± 0.096	5.19 ± 0.91	NR	NR
2 - A549	3000	12.9 ± 1.4	2069 ± 96	45.6 ± 2.2	2966 ± 55			1.75 ± 0.12	6.10 ± 0.64	NR	NR
3 - A549	3000	12.1 ± 1.4	2227 ± 94	38.7 ± 2.1	3259 ± 59			1.90 ± 0.17	5.76 ± 0.58	NR	NR
4 - A549	3000	17.7 ± 1.6	1612 ± 54	86.5 ± 4.5	2598 ± 41	Equal to EC_{50} values		1.38 ± 0.080	4.61 ± 0.28	NR	NR
4 - BEAS-2B	3000	53.5 ± 3.2	1032 ± 54	120.7 ± 4.5	2591 ± 71			2.70 ± 0.23	2.39 ± 0.14	NR	NR
5 - A549	3000	12.9 ± 1.2	2499 ± 76	35.2 ± 1.3	3247 ± 36			2.18 ± 0.17	8.40 ± 0.87	NR	NR
5 - BEAS-2B	3000	7.5 ± 0.70	805 ± 35	18.9 ± 0.88	1221 ± 36			2.37 ± 0.17	5.28 ± 0.65	NR	NR
		YTX	hYTX	YTX	hYTX	YTX	hYTX	YTX	hYTX	YTX	hYTX
6 - A549	8000	1.49 ± 0.40	NA	3.87 ± 0.63	NA	2.49 ± 0.26	NA	3.23 ± 1.30	NA	38.0 ± 2.5	NA
6 - BEAS-2B	8000	0.19 ± 0.13	NA	1.40 ± 0.23	NA	1.03 ± 0.18	NA	1.19 ± 0.41	NA	15.5 ± 3.7	NA
7 - A549	8000	1.19 ± 0.20	NA	1.88 ± 0.14	NA	1.67 ± 0.13	NA	5.55 ± 2.07	NA	24.6 ± 3.2	NA
7 - BEAS-2B	8000	0.81 ± 0.10	NA	1.31 ± 0.11	NA	1.24 ± 0.09	NA	4.88 ± 1.63	NA	12.0 ± 3.1	NA
8 - A549	8000	0.14 ± 0.018	NA	1.45 ± 0.22	NA	0.50 ± 0.04	NA	1.31 ± 0.13	NA	37.6 ± 1.4	NA
8 - BEAS-2B	8000	0.06 ± 0.017	NA	NR (see min.)	NA	0.35 ± 0.06	NA	0.77 ± 0.12	NA	52.2 ± 1.9	NA
9 - A549	3000	0.20 ± 0.063	0.031 ± 0.011	1.65 ± 0.25	0.975 ± 0.38	0.88 ± 0.11	0.267 ± 0.071	1.24 ± 0.22	0.812 ± 0.13	26.8 ± 2.0	32.5 ± 2.3

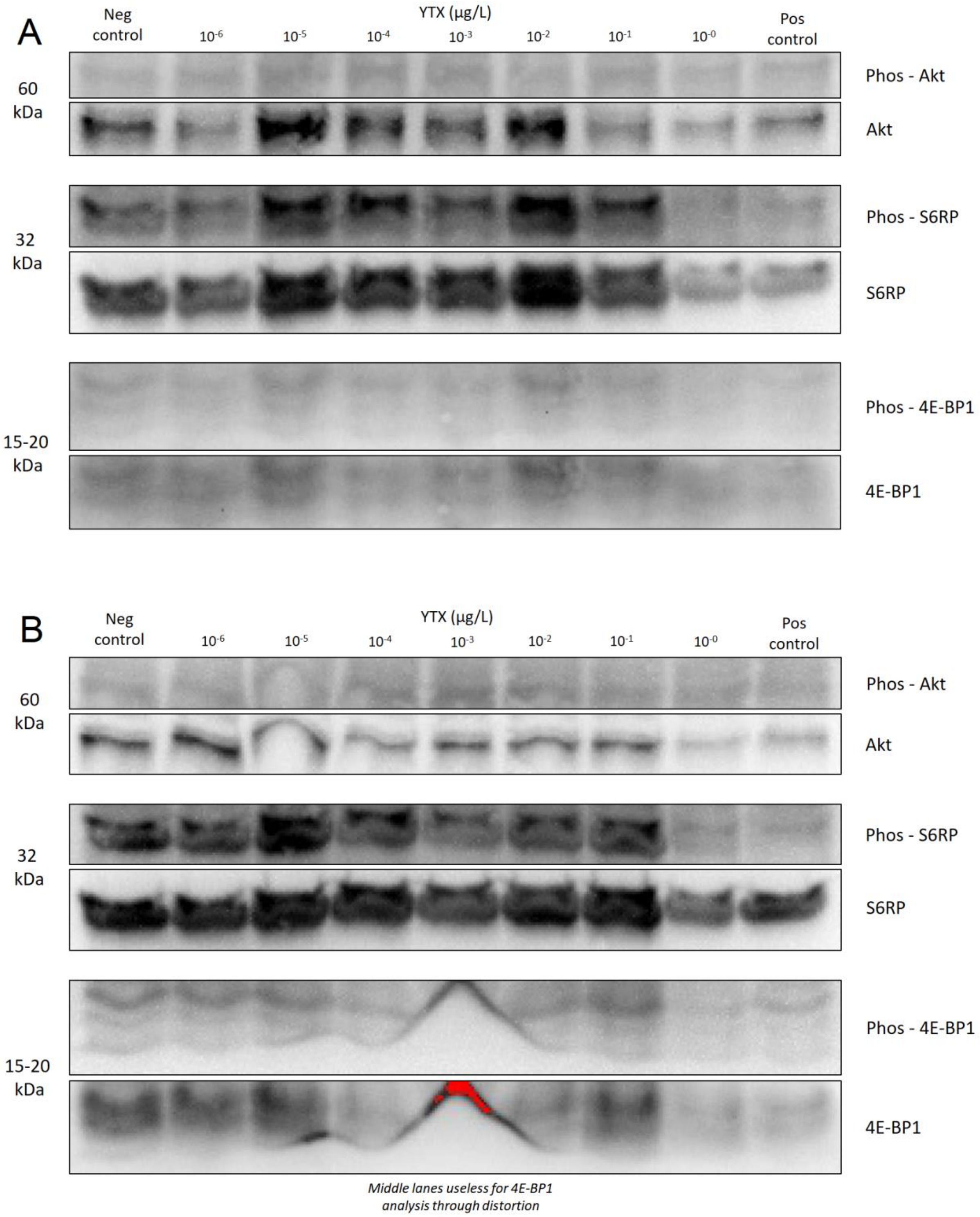

Figure S4: Cropped, non-edited versions of blots related to the experiment shown in Figure 3. For both (A) the A549 and (B) BEAS-2B cell line, a representative example of one of the blots is shown. A darker band indicates a stronger chemiluminescent signal.

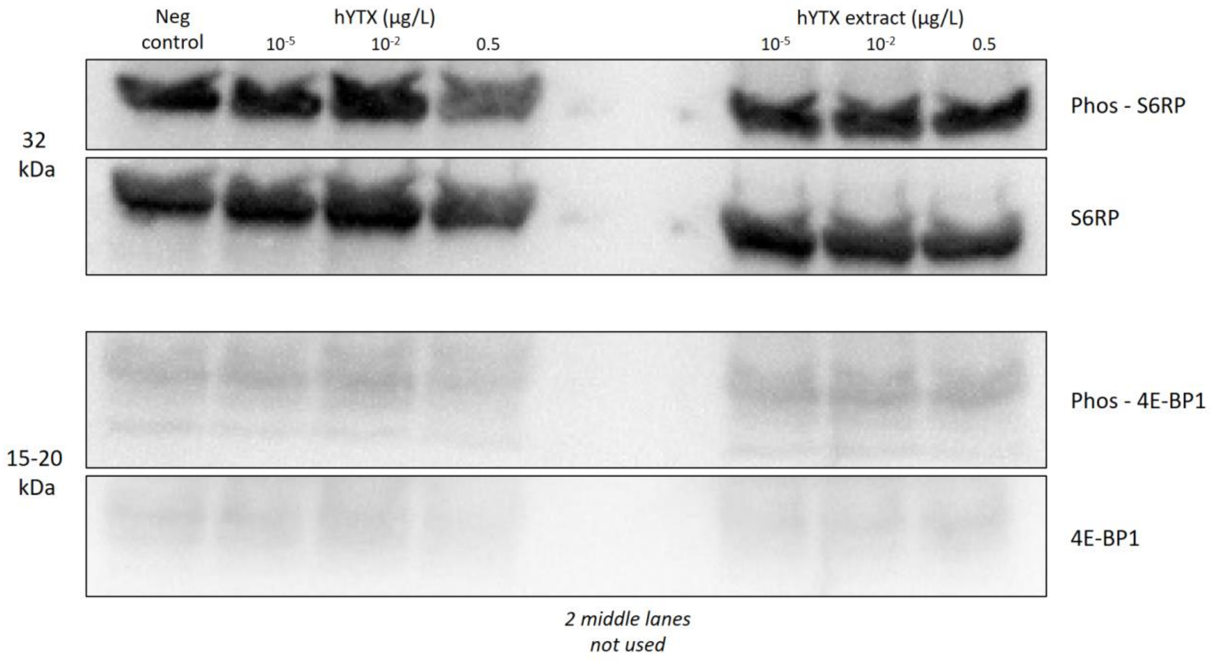

Figure S5: Cropped, non-edited versions of a representative blot for the experiment shown in Figure 4. A darker band indicates a stronger chemiluminescent signal.
